# Is there a route for metachronous inguinal lymph node in colonic cancer? A case report

**DOI:** 10.1093/jscr/rjae024

**Published:** 2024-02-21

**Authors:** Kammoun Neirouz, Trabelsi Mohamed Mehdi, Guelbi Mohamed, Messaoudi Sohaib, Oueslati Annouar, Khalfallah Mehdi, Nouira Ramzi

**Affiliations:** Department B of Surgery, Charles Nicolle Hospital, Tunis 1006, Tunisia; Department B of Surgery, Charles Nicolle Hospital, Tunis 1006, Tunisia; Department B of Surgery, Charles Nicolle Hospital, Tunis 1006, Tunisia; Department B of Surgery, Charles Nicolle Hospital, Tunis 1006, Tunisia; Department B of Surgery, Charles Nicolle Hospital, Tunis 1006, Tunisia; Department B of Surgery, Charles Nicolle Hospital, Tunis 1006, Tunisia; Department B of Surgery, Charles Nicolle Hospital, Tunis 1006, Tunisia

**Keywords:** case report, colonic carcinoma, inguinal lymph nodes, metastasis

## Abstract

As the inguinal lymph nodes do not serve as the primary route for the lymphatic drainage of the colon, inguinal metastasis from colorectal carcinomas is considered an unusual finding, especially in the 2nd year follow-up. A 76-year-old male patient, operated on for non-metastatic right colic adenocarcinoma, consulted 2 years after for a right inguinal swelling. A biopsy was performed. Unexpectedly, it showed an adenocarcinoma metastasis in favor of a colonic origin. There was no relapse of the disease. The pathological examination of the resected inguinal lymph node confirmed malignant cells from a colonic origin. As the positron emission tomography scan showed no other tumoral localizations, a multidisciplinary discussion ensued, culminating in the choice of chemotherapy for optimal pathological response. This case highlights the fact that colic drainage may encounter inguinal lymph nodes and thus inguinal groin metastasis could exceptionally have been seen in colonic carcinomas.

## Introduction

Inguinal lymph node metastasis from colorectal carcinomas is considered an unusual finding [1]. The mechanism of this invasion is not universally well codified. As the superficial pathway originating from the inferior epigastric artery in abdominal wall invasion is the best-known mechanism, there are some other ways, less elaborate in the literature, that can be a route for inguinal lymph nodes in colonic cancer. In the face of this ambiguity, and through our case report, we aim to report a metachronous isolated inguinal lymph node metastasis from an adenocarcinoma of the ascending colon.

## Case presentation

A 76-year-old male patient who was under follow-up for chronic bronchitis was complaining of abdominal pain and bloating. He was explored by an endoscopy with a biopsy revealing a right colic adenocarcinoma. A body computed tomography (CT) scan was performed for the work-up showing right peri-colic nods, with a right colic budding mass. There were no secondary lesions. A surgical approach was chosen, involving a midline incision, as laparoscopy was contraindicated because of the patient’s respiratory distress. Per-operatively, we found an ascending right colon tumor. He had a carcinologic right colectomy with ileo-colic anastomosis. Pathological anatomy of the specimen showed a well-differentiated adenocarcinoma classified pT3N1aM0; 1 of the 29 lymph nodes examined was positive and he had adjuvant chemotherapy. A 1 year-follow-up including a CT scan and colonoscopy showed no recurrence.

**Figure 1:**
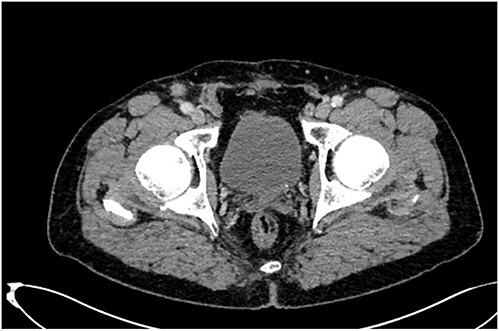
A right inguinal lymph node in a CT scan.

**Figure 2:**
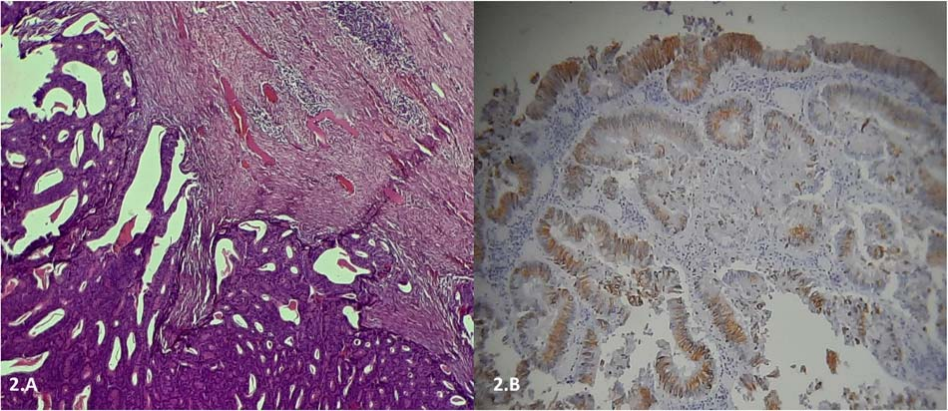
(A) Histological examination, after staining with hematoxylin and eosin, showing a lymph node parenchyma massively invaded by a carcinomatous proliferation consisting essentially of glands and cribriform structures lined by a cylindrical coating with pseudostratified nuclei, the site of moderate to significant cytonuclear atypia with several mitoses. (B) Immunohistochemical study showing a massive expression of cytokeratin 20.

Furthermore, tumor marker levels, notably carcinoembryonic antigen (CEA) and cancer antigen 19-9 (CA 19-9), were normal. At a 2-year follow-up, the patient consulted for a right inguinal swelling. On physical examination, the abdomen was soft with no palpable mass and we found a right groin adenopathy measuring 4 cm. A biopsy was performed. Unexpectedly, it showed an adenocarcinoma metastasis in favor of a colonic origin. A CT scan was performed. There was no relapse of the disease. However, it revealed an enlarged right inguinal lymph node (Fig. 1). A resection of the inguinal lymph node was performed. Histological examination, after staining with hematoxylin and eosin, revealed a lymph node parenchyma massively invaded by a carcinomatous proliferation consisting essentially of glands and cribriform structures lined by a cylindrical coating with pseudostratified nuclei, the site of moderate to significant cytonuclear atypia with several mitoses (Fig. 2A). An immunohistochemical study showed a massive expression of cytokeratin 20 (Fig. 2B). This pathological examination led us to confirm the metastatic nature of the lymph node. As the positron emission tomography (PET) scan showed no other tumoral localizations, a multidisciplinary discussion ensued, culminating in the choice of chemotherapy for optimal pathological response.

## Discussion

Inguinal lymph node invasion in right colon carcinomas is an exceedingly rare occurrence, and its rarity can be attributed to anatomical factors. Typically, inguinal lymph nodes do not serve as the primary route for the lymphatic drainage of the colon. Instead, the colon’s anatomical drainage pathway involves a sequence that includes the epicolic and paracolic lymph nodes, followed by the main nodes situated at the base of the superior and inferior mesenteric arteries [2]. To reach the inguinal nodes, a deviation from this standard route is required, involving the hypogastric pathway and nodes along the external iliac, common iliac, and paraaortic groups. This deviation might help explain the exceptional cases of inguinal metastasis observed in rectal carcinomas. However, when it comes to right colic carcinomas, excluding instances where abdominal wall invasion is involved and could potentially lead to the spread through the hypogastric pathway, there exists no clear anatomical rationale for inguinal metastasis or even iliac lymph node invasion [3]. To the best of our knowledge, this is the first report of a metachronous inguinal metastasis arising from a carcinoma of the right colon without an abdominal wall invasion. A comprehensive review of the existing literature, focused on documented cases, has revealed only six instances of colon adenocarcinoma with inguinal lymph node metastasis [3–8]. In three of these cases [3–5, 7], abdominal wall invasion was evident, and the mechanism of inguinal lymph node metastasis was elucidated by a superficial pathway originating from the inferior epigastric artery and leading to the inguinal nodes. In another case, invasion of the iliac nodes preceded metastasis to the inguinal nodes [6]. The case presented by Hakeem *et al.* [8] shares similarities with our own, in that no straightforward explanation exists for the occurrence of inguinal groin metastasis. Nonetheless, even in comparison with this case, ours remains exceptional due to the unique circumstance where the metastasis manifested a year after follow-up, despite adjuvant chemotherapy and a negative extension work-up.

## Conclusion

Colic carcinomas are not described to give inguinal lymph node invasion. This could be explained by the fact that colic drainage has a cephalic pathway along the mesenteric arteries. However, in some cases, such as in ours, inguinal groin metastasis could exceptionally have been seen.

## Data Availability

Data sharing is not applicable.
